# Epidemiological investigation, related factors, spatial–temporal cluster analysis of pseudorabies virus seroprevalence in Guangdong Province of China

**DOI:** 10.3389/fvets.2025.1581043

**Published:** 2025-06-04

**Authors:** Mengpo Zhao, Jing Chen, Shengjun Luo, Pian Zhang, Jinliang Chen, Chenglong Sun, Zhaowen Ren, Yanju Huang, Xiaoxiao Zhang, Hua Xiang, Yuan Huang, Gang Wang, Zi-Guo Yuan, Xiaohu Wang

**Affiliations:** ^1^College of Veterinary Medicine, South China Agricultural University, Guangzhou, China; ^2^Guangdong Provincial Key Laboratory of Livestock Disease Prevention, Guangdong Provincial Observation and Research Station for Animal Disease, Institute of Animal Health, Guangdong Academy of Agricultural Sciences, Guangzhou, China; ^3^Department of Molecular Biology, University of Texas Southwestern Medical Center, Harry Hines Boulevard, Dallas, TX, United States

**Keywords:** pseudorabies virus, seroprevalence, epidemiological investigation, related factors, spatial–temporal clustering, Guangdong province trainable epidemiological investigation

## Abstract

**Introduction:**

Pseudorabies (PR) is an important zoonotic viral disease that infects a wide range of animals, including humans. In recent years, the prevalence of pseudorabies virus (PRV) has caused great economic losses to the Chinese pig industry.

**Methods:**

In this study, 40,050 serum samples were collected from 348 pig farms in 18 districts of Guangdong Province, China, between 2017 and 2022 to investigate the seroprevalence of wild-type PRV in pigs.

**Results:**

The results of the enzyme-linked immunosorbent assay (ELISA) showed that seropositivity for PRV gE antibodies was 25.28% (95% CI, 24.86% to 25.71%) at the pig level. However, the seropositivity of PRV gE antibodies reached 67.44 % (95% CI, 62.14% to 71.96%) at the farm level. To identify potential factors associated with the positive rate of PRV gE antibodies, logistic regression analysis was performed, and the results showed that the seropositivity rate of PRV gE was related to factors such as geographic distribution and season. To find areas with higher PR prevalence in Guangdong Province, China, we analyzed the data using SaTScan 10.2.5 software and identified five spatiotemporal clusters of higher PRV gE antibody positivity in Guangdong Province, China, with the highest prevalence from April to June 2018.

**Conclusion:**

Our study revealed seroprevalence, associated influencing factors, and spatiotemporal clustering characteristics of PRV gE antibody positivity in Guangdong Province, China, in recent years. This provides new scientific data for the development of policies related to the prevention and control of wild-type pseudorabies epidemics in Guangdong Province, China.

## 1 Introduction

Pseudorabies virus (PRV) is a double-stranded DNA virus in the family Herpesviridae, subfamily *α*-herpesviridae, genus Varicella virus (1). Pseudorabies (PR) also known as Aujeszky’s disease (AD), is an acute, febrile infectious disease common to a wide range of domestic and wild animals caused by PRV infection (2, 3). The disease can infect a wide range of economically farmed and wild animals, and pigs are the natural and reservoir hosts of the disease (4–6). PRV can infect pigs at different stages, with clinical symptoms such as respiratory distress, diarrhea, miscarriage, and even death (7–9).

While PRV has been eradicated in North America and parts of Europe, it remains a major cause of reproductive disorders in sows in China (10). In the 1970s, the PRV Bartha-K61 vaccine strain was introduced into China and widely used for PRV prevention (11). At the end of 2011, there was a widespread PR epidemic in Chinese pig farms, where PRV variant strains (JS-2012, TJ and FJ strains, etc.) were mutated in several genes, allowing them to evade protection from traditional vaccines and be highly pathogenic to piglets and sows (12, 13). Up to now, mutant PRV is still prevalent in pig farms in China and causes more serious symptoms (14, 15), which seriously threatens the healthy development of China’s livestock farming industry.

Due to the increasing prevalence of wild-type PRV, enzyme-linked immunosorbent assay (ELISA) method based on PRV gE gene is usually used to distinguish PRV vaccine strains (gE gene deleted) from naturally infected strains (16), Therefore, timely PR serological investigation is essential to prevent PR epidemic and outbreak. Following the outbreak of African swine fever, most pig farms have adopted stricter biosecurity controls, which makes it more difficult to collect blood from pig farms and impossible to accurately estimate the number of pigs in the study area. The outbreak of African swine fever (ASF) has had a huge impact on the pig industry and has prompted farms to step up their biosecurity measures. These measures have not only targeted African swine fever but have also affected the spread of PRV to some extent. In addition, there are no more specific and relevant data on the reporting of pseudorabies seroprevalence, associated factors, and spatial and temporal analyses in Guangdong Province, China, after 2020. Therefore, in this study, 40,050 pig blood samples were collected from 348 pig farms in 18 districts of Guangdong Province, China, using a convenient sampling plan to test for PRV gE antibody positivity from 2017 to 2022. The geographic location of PRV gE antibody-positive farms can help to identify areas of high prevalence of wild-type PRV. This information may provide more accurate and effective measures for swine pseudorabies prevention and control in Guangdong Province, China.

## 2 Materials and methods

### 2.1 Study area

The area studied is from 109°45′ to 117°20′E longitude and from 20°09′ to 25°31′N latitude, with an area of approximately 17,977 square kilometers. From 2017 to 2022, a total of 40,050 blood samples were collected from 348 pig farms in 18 regions of Guangdong Province, China, covering four areas including Eastern Guangdong (Shantou, Chaozhou, Jieyang, and Shanwei), Western Guangdong (Zhanjiang, Maoming, and Yangjiang), Northern Guangdong (Shaoguan, Qingyuan, Yunfu, Meizhou, and Heyuan), and Pearl River Delta (Guangzhou, Foshan, Dongguan, Zhaoqing, Jiangmen, and Huizhou). In addition, the location coordinates of the pig farm were obtained from Baidu Maps.^1^

### 2.2 Sample collection

All adult sows are vaccinated with live PR vaccine (Bartha-K61 strain) and inactivated vaccine (Bartha K61 strain) every 4 months. Gilts are given intramuscular injections at about 6 months of age, followed by booster immunization at intervals of 1 month, and another immunization at about 1 month before delivery. Boars are immunized once a year in spring and autumn. Piglets should receive intranasal or intramuscular immunization at 1–3 days of age after birth, and booster immunization should be administered every 2 months. The immunization program for growing pigs is to immunize once at 2–3 months of age and strengthen immunization once at about 4 months of age. The gE gene is naturally deleted in PRV (Bartha-K61 strain) and is carried by wild-type PRV.

Depending on the size of the farm, 5–10, 11–60, and 61–90 samples were collected per small (< 500 pigs), medium (500–2,000 pigs), and large (> 2,000 pigs). Collect 3 to 5 milliliters of blood from the anterior vena cava of pigs using sterile needles or vacuum blood collection tubes. The collected blood is then transported to the laboratory via cold chain transport. Subsequently, centrifuge the blood at 3,000 rpm/min for 10–15 min. Transfer the supernatant serum to a sterile centrifuge tube. All animal handling processes comply with international regulations and animal welfare requirements. All serum samples were collected and stored at −20°C, and detailed information on each sample, including location, collection date, and farm size, was recorded.

We use the online tool epitools to calculate the sample size at herd level, and the minimum sample size is 324 pig farms. Then we use the following formula to calculate the number of animals sampled from each pig farm:


$$n=(1-{\left(\alpha \right)}^{1/D})(N-\frac{D-1}{2})$$

where n is the required sample size, a is the value of 1 minus the confidence level of disease prevalence, D is the estimated minimum number of diseased animals in the pig farm, and N is the animal size. This requires a minimum sample size of 28 per pig farm. If the total number of pigs raised on the farm is less than 28, serum will be collected from all pigs.

### 2.3 Serological detection

PRV gE antibodies were detected in 40,050 serum samples using a commercial ELISA kit (Cat: CP144, IDEXX Laboratories, Westbrook, ME). The presence of anti-PRV gE antibodies was determined by calculating the S/N (absorbance of serum wells versus negative control wells) ratio for each sample. Samples with S/N ≦ 0.60 were considered positive for wild PRV infection, while those with S/N > 0.70 were negative. Samples with 0.6 < S/N ≦ 0.70 are considered suspect and require additional testing or repeated testing over time to determine if the sample is negative or positive.

### 2.4 Statistical analysis

All collected data was inputted and calculated using Microsoft Excel 2021, a spreadsheet software developed by Microsoft in the United States. The farm is considered positive for wild-type PRV infection if at least one farm serum sample is positive for the PRV gE antibody. If no PRV gE antibody is detected, the farm is considered a negative farm that is not infected with wild-type PRV. The logistic regression model in SPSS 26.0 software (IBM, Chicago, IL, United States) was used to analyze the correlation between serum prevalence of PRV gE antibody and various factors such as time, region, season, and pig population. Calculate the positive rate and 95% confidence interval of serum prevalence of PRV gE in pig herds. In this study, statistical significance was determined by a *p* value.

Using the Bernoulli model (17, 18) of SaTScan 10.2.5 software to predict the spatiotemporal clustering distribution of high serum prevalence of PRV gE. Time clustering analysis is conducted at the monthly level, covering the sample collection phase from January 2017 to December 2022. In addition, map creation was facilitated through the utilization of ArcGIS Pro software developed by ESRI, United States.

## 3 Results

### 3.1 Seroprevalence of PRV in Guangdong Province

**Figure 1:**
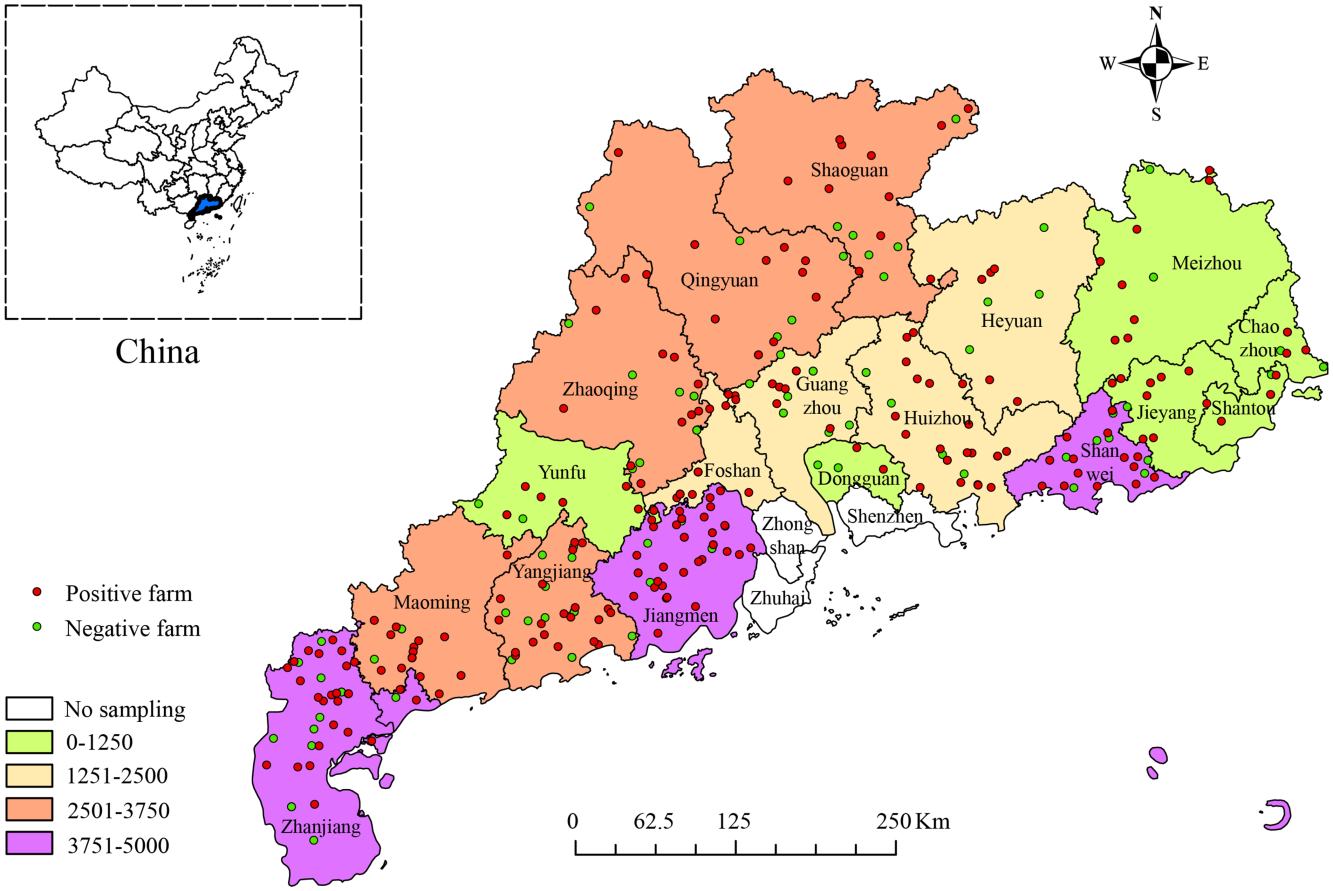
The number of serum samples collected from 18 districts in Guangdong Province, China, and the geographic locations of PRV gE antibody-positive pig farms between January 2017 and December 2022. Different colored boxes represent the number of samples, green dots indicate PRV gE antibody negative farms and red dots indicate PRV gE antibody positive farms.

**Figure 2:**
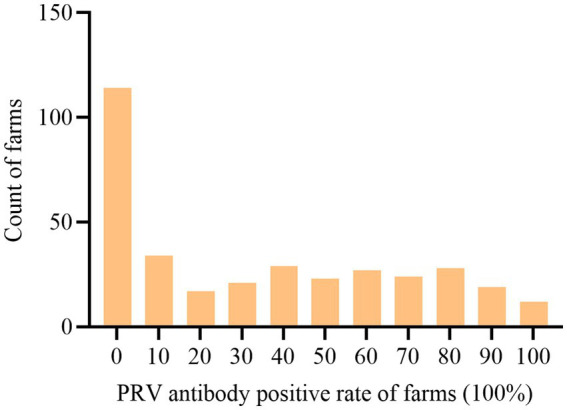
Proportion of pig farms with different positive rates of PRV gE antibody. Samples were collected from January 2017 to December 2022, and PRV seropositivity rates were determined for each of the 348 pig farms. The number of pig farms in each range of positive PRV gE antibodies was then calculated from 0 to 100% in 10% increments. These data were used to create a histogram with the horizontal axis representing the range of positivity rates and the vertical axis representing the number of pig farms.

Between January 2017 and December 2022, 40,050 blood samples from pigs at different stages of life were collected from 348 pig farms in 18 districts of Guangdong Province, China (Figure 1). Based on the statistical data analyzed in this study, at the individual pig level, the positive rate of PRV gE antibody was 25.28% (10,125/40,050, 95% CI, 24.86 to 25.71%) among all serum samples, with significant differences in the positive rates of PRV gE antibody among different regions. At the farm level, the proportion of positive farms (number of samples positive for PRV gE antibodies ≥1) was 67.44% (234/348, 95% CI, 62.14 to 71.96%), with significant differences in the proportion of positive farms in different regions (Table 1). In addition, the positive rate of PRV gE antibodies on farms ranged from 0 to 100% (Figure 2).

**Table 1:** Positive rates of PRV gE antibodies determined by Pearson’s chi-square test in each regional province of Guangdong.

Regular area	Regions	Samples^a^			Pig farms^b^		
		No. of positive samples	Total no. of sample	Seroprevalence rate (%; 95% CI)	No. of positive farms	Total no. of farms	Farm positivity rate (%; 95% CI)
Eastern Guangdong	Shantou	163	545	29.91 (26.22–33.88)	4	6	66.67 (30.11–90.32)
	Chaozhou	45	809	5.56 (4.18–7.36)	4	8	50.00 (17.45–82.55)
	Jieyang	433	971	44.59 (41.49–47.73)	6	8	75.00 (40.93–92.58)
	Shanwei	1,033	4,967	20.80 (19.69–21.95)	13	22	59.10 (38.73–76.74)
Western Guangdong	Zhanjiang	698	4,354	16.03 (14.97–17.15)	29	43	67.44 (52.51–79.51)
	Maoming	817	2,071	39.45 (37.37–41.57)	19	23	82.61 (62.86–93.02)
	Yangjiang	869	3,704	23.46 (22.12–24.85)	21	37	56.76 (40.92–71.33)
Northern Guangdong	Shaoguan	296	3,003	9.86 (8.84–10.98)	10	17	58.82 (36.45–78.39)
	Qingyuan	644	2,699	23.86 (22.29–25.50)	11	18	61.11 (38.62–79.69)
	Yunfu	278	1,049	26.50 (23.92–29.25)	11	13	84.62 (57.77–95.68)
	Meizhou	239	841	28.42 (25.48–31.56)	9	12	75.00 (46.77–91.11)
	Heyuan	590	1,484	39.76 (37.30–42.27)	7	11	63.64 (35.38–84.84)
Pearl River Delta	Guangzhou	651	1988	32.75 (30.72–34.84)	11	22	50.00 (30.72–69.28)
	Foshan	414	1,540	26.88 (24.73–29.15)	13	13	100.00 (77.19–100.00)
	Dongguan	78	618	12.62 (10.23–15.47)	2	5	40.00 (11.76–76.93)
	Zhaoqing	842	2,998	28.09 (28.05–30.66)	13	25	52.00 (33.75–69.97)
	Jiangmen	1,367	4,659	29.34 (27.61–31.09)	31	39	79.49 (64.47–89.22)
	Huizhou	668	1,750	38.17 (35.92–40.47)	20	26	76.92 (57.95–88.96)
Total		10,125	40,050	25.28 (24.86–25.71)	234	348	67.44 (62.14–71.96)

a The chi-square test value for the rate of positive swine serum PRV gE antibodies in different regions was 7,336.25, ***p < 0.001.

b The chi-square test value for the rate of positive pig farms in different regions was 65.82, ***p < 0.001.

### 3.2 Seroprevalence of PRV gE antibodies in different regions of Guangdong Province

At the individual swine level, the regions with higher seropositivity rates were Jieyang, Heyuan, Maoming, and Huizhou, with PRV gE antibody positivity rates of 44.59% (95% CI, 41.49 to 47.73%), 39.76% (95% CI, 37.30 to 42.27%), 39.45% (95% CI, 37.37 to 41.57%), and 38.17% (95% CI, 36.28 to 40.15%). In contrast, 9.86% (95% CI, 8.84 to 10.98%) of Shaoguan and 5.56% (95% CI, 4.18–7.36%) of Chaozhou serum samples were less than 10% positive for PRV gE antibodies. Pearson’s chi-square test for seropositivity to PRV gE showed significant differences in seropositivity rates between regions in Guangdong Province (from 5.66 to 44.59%), with *p*<0.001 (Table 1).

At the pig farm level, Foshan, Yunfu, and Maoming had the highest positive rates of PRV gE antibody at 100% (95% CI, 77.19 to 100.00%), 84.62% (95% CI, 57.77–95.68%) and 82.61 (95% CI, 65.06 to 90.23%), respectively. In contrast, 40.00% (95% CI, 62.86–93.02%) of serum samples from Dongguan had the lowest PRV gE antibody positivity rate. The results of Pearson’s chi-square test for PRV gE seropositivity showed that the number of farms in Guangdong province where serum samples were detected as positive varied significantly among the various regions of Guangdong province (40.00 to 100.00%), with *p*<0.001 (Table 1).

The positive rate of PRV gE antibody in serum samples from the Pearl River Delta was highest, at 29.66% (95% CI, 28.89 to 30.43%). Results showed that the positive rate of PRV gE antibody in Guangdong Province decreased from 33.15% (95% CI, 32.18 to 34.25%) to, 10.56% (95% CI, 9.59 to 11.62%) from 2017 to 2022 (Table 2). The positive rate of PRV gE antibody was the lowest in serum samples from northern Guangdong at 22.55% (95% CI, 21.70 to 23.42%). There was a significant difference in serum positivity rates between the Pearl River Delta and eastern Guangdong, western Guangdong, and northern Guangdong (chi-square test, *p* < 0.001; Table 2).

**Table 2:** Pearson’s chi-square test for factors associated with PRV serological status at the sample level.

Factor	Category	No. positive	No. sample	Seroprevalence rate (%; 95% CI)
Year^a^	2017	2,925	8,824	33.15 (32.18–34.25)
	2018	3,371	10,276	32.80 (31.89–33.71)
	2019	2,618	11,636	22.50 (21.75–23.27)
	2020	528	3,359	15.72 (14.53–16.99)
	2021	311	2,433	12.78 (12.51–14.17)
	2022	372	3,522	10.56 (9.59–11.62)
Regions^b^	Pearl River Delta	4,020	13,553	29.66 (28.89–30.43)
	Eastern Guangdong	1,674	7,292	22.96 (22.01–23.94)
	Western Guangdong	2,384	10,129	23.54 (22.72–24.38)
	Northern Guangdong	2,047	9,076	22.55 (21.70–23.42)
Pig herd^c^	Sows	524	3,985	13.15 (12.14–14.23)
	Boars	1,186	4,241	27.97 (26.64–29.34)
	Gilts	873	5,236	16.67 (15.68–17.71)
	Piglets	3,383	11,170	30.29 (29.44–31.15)
	Nursery pigs	1,287	4,452	28.91 (27.59–30.26)
	Fattening pigs	2,872	10,966	26.19 (25.38–27.02)
Season^d^	Spring	2,456	10,367	23.69 (22.88–24.52)
	Summer	3,189	10,690	29.83 (28.97–30.71)
	Autumn	2,199	9,239	23.80 (22.94–24.68)
	Winter	2,281	9,754	23.39 (22.56–24.24)

a The chi-square test value for PRV gE seroprevalence in different years was 962.33. ***p < 0.001.

b The chi-square test value for PRV gE seroprevalence in different regions was 563.94. ***p < 0.001.

c The chi-square test value of PRV gE seroprevalence in different seasons was 835.56. ***p < 0.001.

d The chi-square test value of PRV gE seroprevalence in different pig stages was 371.38. ***p < 0.001.

### 3.3 Serum prevalence of PRV gE antibodies in pig herds at different stages

**Figure 3:**
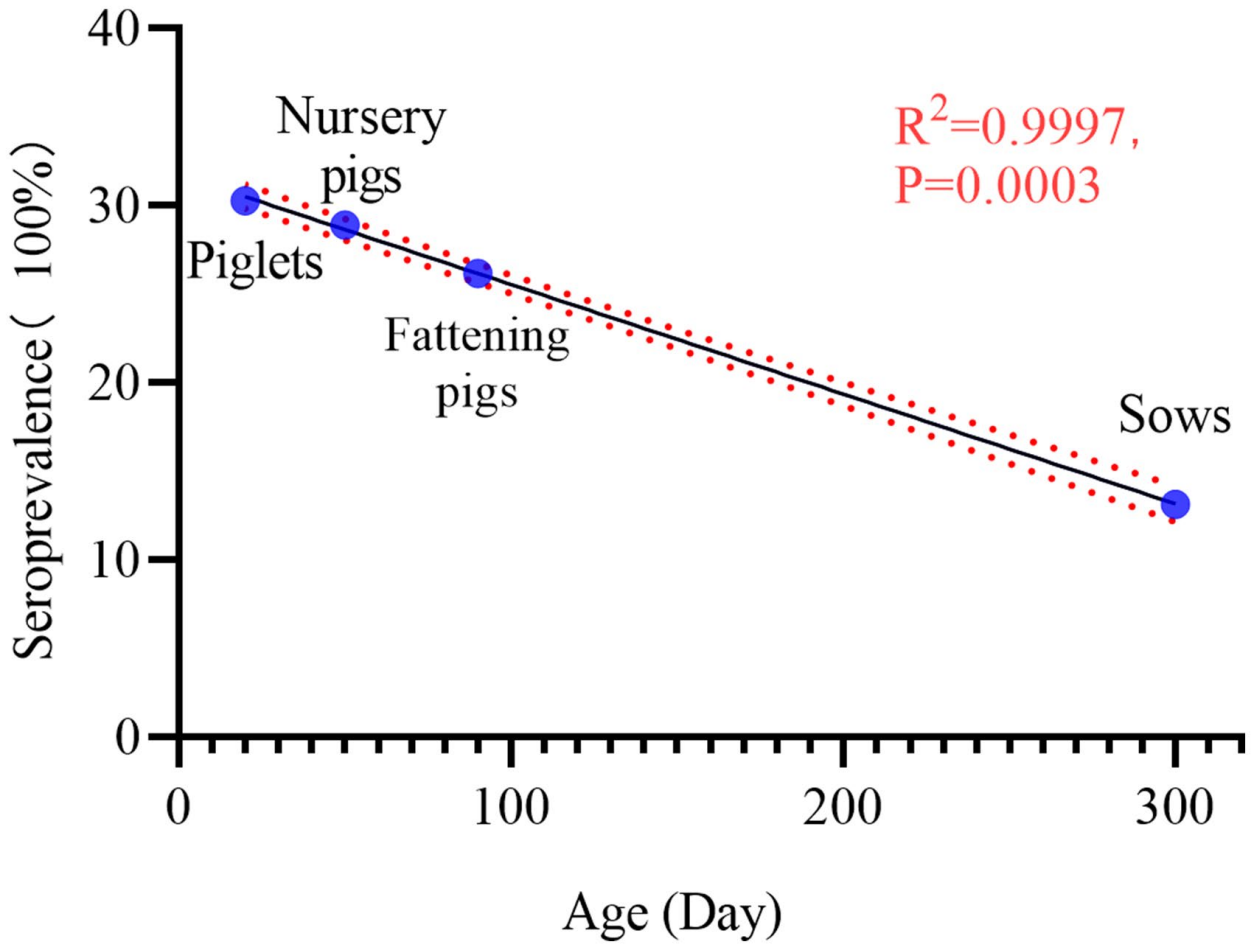
Seroprevalence of PRV gE in pigs at different stages. Seropositivity decreased linearly from piglets, Nursery pigs, fattening pigs to sows: 30.29% (95% CI, 29.44 to 31.15%), 28.91% (95% CI, 27.59 to 30.26%), 26.19% (95% CI, 25.38 to 27.02%), 13.15% (95% CI, 12.14–% to 14.23). The R2 value of its trendline was 0.9997, *p* = 0.0003.

The results of PRV gE sera antibody tests collected from sows, boars, gilts, piglets, nursery pigs, and fattening pigs were classified and counted. The results are shown in Table 2. Serum positivity for PRV gE antibodies was highest at 30.29% (95% CI, 29.44 to 31.15%) in the piglet group and lowest at 13.15% (95% CI, 12.14 to 14.23%) in the sow group. In addition, RV gE antibody positivity was significantly lower in sows than in gilts, gilts, piglets, nursery pigs, and fattening pigs (chi-square test, *p* < 0.001). Interestingly, the results of rate fitting curves for piglets (20 days), fattening pigs (50 days), fat pigs (90 days), and sows (300 days) showed a linear decrease in RV gE antibody positivity from piglets to sows (Figure 3).

### 3.4 Seasonal levels of seroprevalence of PRV-gE antibody

We classified and counted PRV gE antibody detection results in pig sera collected during different seasons. Results showed that PRV gE antibody positivity was highest at 29.83% (95% CI, 28.97 to 30.71%) of pig sera collected during the summer and lowest at 23.39% (95% CI, 22.56 to 24.24%) of pig sera collected during the winter. In addition, PRV gE antibody positivity was significantly higher in summer than in spring, fall, and winter (chi-square test, *p* < 0.001; Table 2).

### 3.5 Investigation of factors associated with a positive rate of PRV gE antibody

The Pearson chi-square test showed that the *p*-values of factors such as region, herd, and season were less than 0.001. Therefore, these factors were included in the Univariate Logistic Analysis model. Univariate logistic analysis identified three factors associated with the positive rate of PRV gE antibodies (Table 3). Compared with the Pearl River Delta region, pigs in eastern Guangdong, western Guangdong, and northern Guangdong were significantly less likely to be infected with PRV, with odds ratios of (OR, 0.71; 95% CI, 0.66 to 0.76%), (OR, 0.73; 95% CI, 0.69 to 0.78%) and (OR, 0.69; 95% CI, 0.65 to 0.73%). Boars (OR, 2.56; 95% CI, 2.29 to 2.88%), gilts (OR, 1.32; 95% CI, 1.18 to 1.49%), piglets (OR, 2.87; 95% CI, 2.60 to 3.17%), Nursery pigs (OR, 2.69; 95% CI, 2.40 to 3.01%), and fattening pigs (OR, 1.99; 95% CI, 1.83 to 2.17%) had significantly higher rates of PRV infection than sows. In addition, PRV gE seropositivity was significantly lower in spring (OR, 0.73; 95% CI, 0.69 to 0.78%), autumn (OR, 0.74; 95% CI, 0.69 to 0.78%), and winter (OR, 0.72; 95% CI, 0.68 to 0.76%) than in summer. In addition, pigs were more likely to be infected with PRV in summer than in spring, Autumn, and winter.

**Table 3:** Univariate logistic analysis of risk factors associated with serological status of PRV in pig farms.

Factor	Category	OR (95% CI)	*p*-value
Regions	Pearl River Delta	1 (Reference)	
	Eastern Guangdong	0.71 (0.66–0.76)	<0.01
	Western Guangdong	0.73 (0.69–0.78)	<0.01
	Northern Guangdong	0.69 (0.65–0.73)	<0.01
Pig herd	Sows	1 (Reference)	
	Boars	2.56 (2.29–2.88)	<0.001
	Gilts	1.32 (1.18–1.49)	<0.01
	Piglets	2.87 (2.60–3.17)	<0.001
	Nursery pigs	2.69 (2.40–3.01)	<0.01
	Fattening pigs	1.99 (1.83–2.17)	<0.01
Season	Summer	1 (Reference)	
	Spring	0.73 (0.69–0.78)	<0.001
	Autumn	0.74 (0.69–0.78)	<0.001
	Winter	0.72 (0.68–0.76)	<0.001

### 3.6 Spatial–temporal cluster of high serum prevalence of PRV gE

**Figure 4:**
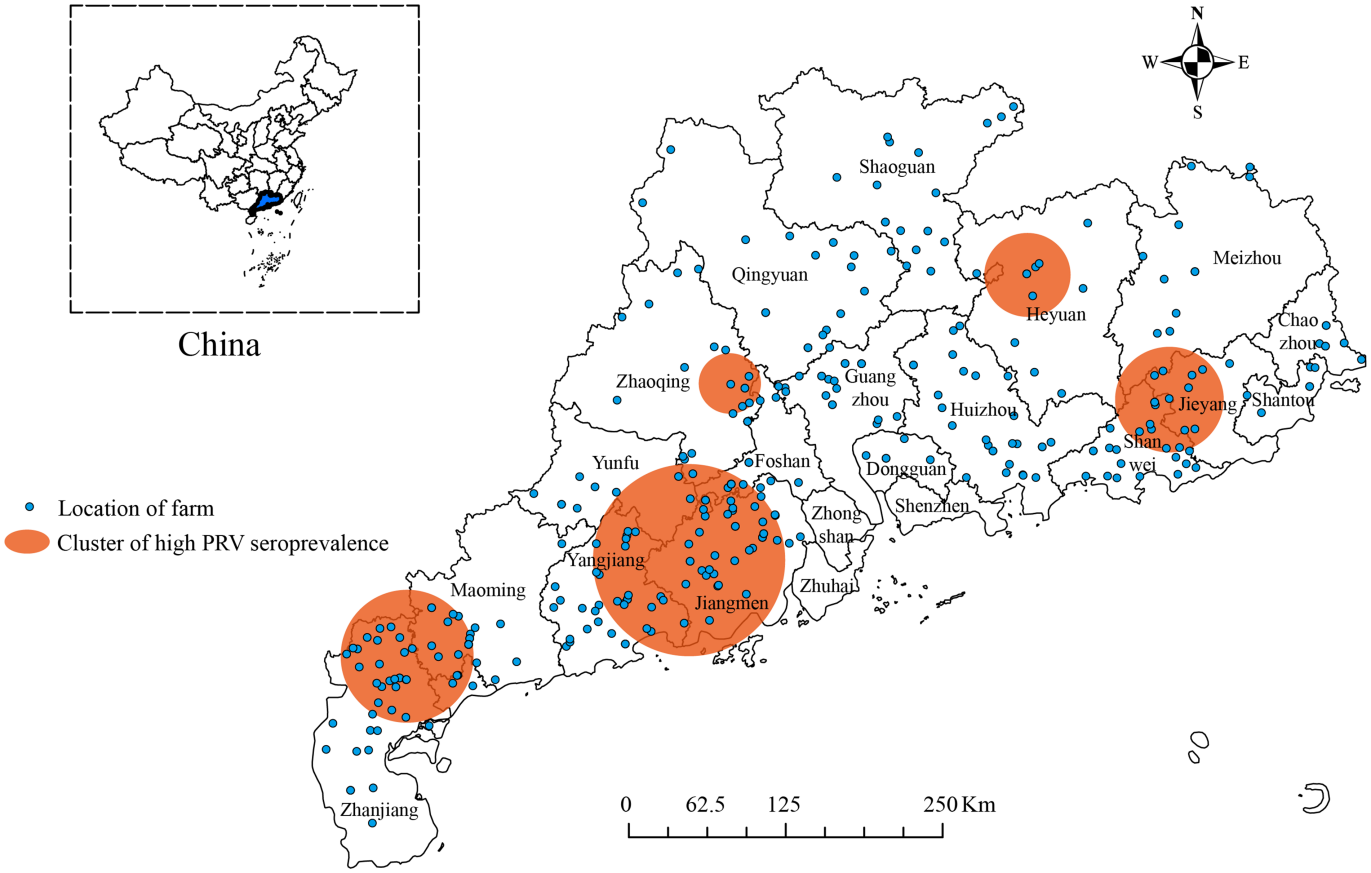
Significant spatial–temporal clusters of high seroprevalence of PRV gE were observed in Guangdong Province, China from January 2017 to December 2022. The blue dots indicate the geographic location of the pig farm. The orange circle represents the region with a higher positive rate for PRV gE antibodies.

The analysis showed that from January 2017 to December 2022, high seroprevalence of PRV gE was found in five clusters in China (Figure 4; Table 4). The first cluster is located at 110.162411E, 21.577763 N radius 83.7 kilometers. It has a relative risk value of 3.13 and a log-likelihood ratio (LLR) value of 1,025.73 as of 2017/2/1–2018/5/31. The second cluster is located at 112.284936E, 22.407795 N with a radius of 119.4 kilometers. It occurred from 2017/6/1 to 2019/9/30 with a relative risk value of 3.41 and an LLR of 508.35. The third cluster is the first large region with a radius of 31.7 kilometers and is located at 112.501167E, 23.557617 N. It runs from 2018/1/1–2018/7/31. The relative risk value was 2.78 and the LLR value was 623.38. The fourth cluster is located at coordinates 114.837624E, and 24.301724 N, and covers an area with a radius of 53.8 kilometers. The cluster covers the period 2018/4/1–2019/9/30. The relative risk value was found to be 3.35, while the LLR value was calculated to be 472.64. The fifth cluster is located at geographic coordinates 116.015051E, 23.794848 N, and has a radius of 64.5 kilometers. The time frame for this cluster is 2018/6/1–2019/4/30. The relative risk value was 2.58 and the likelihood ratio value was 389.73.

**Table 4:** Spatial–temporal clusters of PRV gE seroprevalence in Guangdong Province, China from 2017 to 2022.

Cluster	Coordinates	Cluster radius (km)	Time range (yr/mo/day)	Relative risk	Log likelihood ratio	*p*-value
1	110.162411E, 21.577763 N	83.7	2017/2/1–2018/5/31	3.13	1,025.73	<10^−17^
2	112.284936E, 22.407795 N	119.4	2017/6/1–2019/9/30	3.41	508.35	<10^−17^
3	112.501167E, 23.557617 N	31.7	2018/1/1–2018/7/31	2.78	623.38	<10^−17^
4	114.837624E, 24.301724 N	53.8	2018/4/1–2019/9/30	3.35	472.64	<10^−17^
5	116.015051E, 23.794848 N	64.5	2018/6/1–2019/4/30	2.58	389.73	<10^−17^

## 4 Discussion

Despite China’s great determination to eradicate pseudorabies, genetic recombination between PRV vaccine strains and wild strains has occurred since 2011, resulting in a significant increase in the virulence of emerging recombinant strains, which is a serious threat to China’s pig farming industry (19, 20). In addition, although the positive rate of PRV gE antibodies in China showed a decreasing trend between 2016 and 2021, the overall positive rate of PRV gE antibodies remained around 20% (20–23).

In this study, we collected blood samples from pigs in different areas and stages in Guangdong Province from January 2017 to December 2022 wild-type PRV infection in pig farms by testing for PRV gE antibodies (22). We analyzed 40,050 serum samples from 348 pig farms in 18 regions of Guangdong Province. Antibody-positive PRV gE farms in Guangdong Province were identified, and factors related to the seroprevalence status of PRV gE were successfully identified. The results of the survey showed that 40,050 swine serum samples were positive for PRV gE antibodies at 25.28% (10,125/40,050, 95% CI, 24.86 to 25.71%), and the prevalence of positivity for anti-PRV gE bodies declined from 22.5 to 12.78 from 2019 to 2021. This is in line with the results of Chen et al. (23) investigated the survey in Henan Province from 2019 to 2021, They observed a decrease in the prevalence of PRV gE antibodies from 25 to 16.69%. Positivity at the farm level was 67.44% (234/348, 95% CI, 62.14 to 71.96%). Xia et al. (24) reported 67.6% (95% CI, 57.0–77.0%) positive PRV gE antibodies in swine farms. In addition, Lin et al. (25) showed a positive rate of PRV gE antibody positivity of 23.55% (4,271/18,138, 95% CI, 22.9–24.2%) in a PRV serology survey conducted in Hunan Province from 2016 to 2020. These studies suggest that PRV decontamination studies in China are still challenging.

Pearson’s chi-square test results showed that the antibody positivity rate of swine serum samples collected in summer was 29.83% (95% CI, 28.97–30.71%), significantly higher than in spring, fall, and winter. This result of the highest summer positive rate is consistent with the findings of Wenchao Gao et al. (26). Their study resulted in the highest seropositivity rate of 14.77% (6,203/42,005, 95% confidence interval 14.43–15.11%) in summer. The difference in the positive rate was due to Wenchao Gao, who only collected nationwide serum samples in 2022 in their study. In addition, Zhao et al. (27) found by regression analysis that the summer OR of 1.095 (95% CI, 0.658–1.830) for pig farms was 1.09 times higher than that for fall pig farms (Reference) after the spring and winter OR of <1.00. These results suggest that China is more likely to have a summer outbreak of swine pseudorabies.

In addition, we also noted that the PRV gE antibody positivity rate was 22.50% (95% CI, 21.75–23.27%) in 2019 and significantly decreased to 15.72% (95% CI, 14.53–16.99%) in 2020. This differs from the 2016–2020 PRV serologic survey study in Hunan Province, where PRV gE antibody positivity was 24.86% (95% CI, 23.5–26.2%) in 2019 and 25.46% (95% CI, 24.2–26.8%) in 2020 (5). This phenomenon may be caused by the fact that some farmers may prefer to collect samples from sick or weak pigs after the epidemic of African swine fever in China. We used a fitted curvilinear equation to analyze the relationship between different pig stages and positive rates of PRV gE antibodies. The results showed that the positive rate of RV gE antibody decreased linearly from piglets to sows (R^2^ = 0.9997), and the seropositivity rate of piglets was significantly higher at 30.29% (95% CI, 29.44–31.15%) than that of sows at 13.15% (95% CI, 12.14–14.23%). This is because PRV-infected sows can pass maternal antibodies to their offspring via colostrum, which lasts 12–14 weeks in piglets (28). PRV can enter pigs through the respiratory and digestive tracts, and sows can transmit maternal antibodies to piglets through vertical transmission (29–32). Our survey shows that the serum positivity rate of piglets is 30.29% (95% CI, 29.44–31.15%), significantly higher than that of sows at 13.15% (95% CI, 12.14–14.23%). The Odds Ratio (OR) of piglets relative to infected sows was 2.87 (2.60 to 3.17%), with a *p*-value <0.001. It is worth noting that the correlation analysis of the positive rate of pig serum at different stages shows that the positive rate of pig serum gradually decreases with age, indicating that current PR prevention and control strategies can effectively prevent PRV infection in sows and boars. However, due to the digestive, respiratory, and vertical transmission capabilities of the pseudorabies virus, the serum positivity rate of piglet populations is significantly higher than that of sows and boars. Therefore, more effective strategies need to be developed to better protect piglets from wild-type PRV infection.

For spatiotemporal clustering analysis of serum positive PRV rates, Allepuz et al. (33, 34) found that from 2003 to 2007, a large number of negative sow farms in some areas of Spain turned positive, while some positive sow farms in other areas turned negative. This geographic relationship may support local transmission of PRV. Therefore, the elimination of this disease seems to have spatial components. Berke et al. (35) surveyed 482 farms in Germany, 186 of which were classified as positive. Two high-risk areas were identified through cluster analysis (relative risk = 2.4 and 3.3). The spatial relative risk function is approximated by the prevalence ratio defined by the ratio of local prevalence to the overall prevalence of farms outside the cluster area. The corresponding approximate relative risk map displays and quantifies a clear spatial pattern of disease occurrence frequency. Zhao et al. (27) first detected five high-risk areas with wild-type PRV seroprevalence in China from 2017 to 2021. Due to the possible link between PRV infection and geography, we analyzed the spatiotemporal clustering of serum prevalence of PRV gE in Guangdong Province, China, and identified five significant clusters from January 2017 to December 2022. Compared with the findings of Zhao and Gao (26, 27), we narrowed down and pinpointed the cluster areas with high PRV gE seroprevalence in Guangdong Province, China. PR prevention and control measures can be more carefully formulated for local areas in China. In this study, a large-scale seroepidemiologic survey was conducted between 2017 and 2022, with an overall seropositivity rate of 25.28%. This result represents a cumulative estimate over a five-year period and may not reflect the current risk profile. Therefore, temporal modeling and time-stratified risk estimates will be conducted in future studies to analyze the temporal trajectory of the disease in more detail. These analyses will help policymakers better understand the dynamics of the disease and develop more targeted interventions.

## 5 Conclusion

In this study, we investigated the seroepidemiology of PRV gE from January 2017 to December 2022 in Guangdong Province. We collected 40,050 blood samples from 348 pig farms in 18 districts. All samples were then tested for PRV gE antibodies by competitive ELISA. We found that the seropositivity of PRV gE in Guangdong Province, China, was highest in the summer months. The overall seroprevalence of PRV gE was 25.28% (10,125/40,050, 95% CI, 24.86 to 25.71%) and 67.44% (234/348, 95% CI, 62.14 to 71.96%) at animal and farm levels, respectively. In addition, we analyzed the factors associated with the seroprevalence of PRV gE using one-way logistic regression and found that the geographic location of the farm, herd type, and season could significantly influence the seroprevalence of PRV gE. During the research period from January 2017 to December 2022, five spatial clusters with high PRV gE serum flow rates were identified in Guangdong, China. In conclusion, our findings complement the information on seroprevalence, associated factors, and geographic locations of positive pig farms in Guangdong Province, China, in recent years, and provide a reference for the development of scientific and effective prevention and control measures against PRV epidemics in Guangdong Province, China.

## Data Availability

The original contributions presented in the study are included in the article/supplementary material, further inquiries can be directed to the corresponding authors.

## Footnotes

^1^ https://api.map.baidu.com/lbsapi/getpoint/index.html
